# Clinical 3D modeling to guide pediatric cardiothoracic surgery and intervention using 3D printed anatomic models, computer aided design and virtual reality

**DOI:** 10.1186/s41205-022-00137-9

**Published:** 2022-04-21

**Authors:** Reena M. Ghosh, Matthew A. Jolley, Christopher E. Mascio, Jonathan M. Chen, Stephanie Fuller, Jonathan J. Rome, Elizabeth Silvestro, Kevin K. Whitehead

**Affiliations:** 1 Division of Pediatric Cardiology, Children’s Hospital of Philadelphia, 3401 Civic Center Blvd, Philadelphia, 19104 PA USA; 2 Department of Anesthesia and Critical Care, Children’s Hospital of Philadelphia, Philadelphia, PA USA; 3 Division of Cardiothoracic Surgery, Children’s Hospital of Philadelphia, Philadelphia, PA USA; 4 Division of Cardiovascular and Thoracic Surgery, West Virginia University School of Medicine, Morgantown, WV USA; 5 Department of Radiology, Children’s Hospital of Philadelphia, Philadelphia, PA USA

**Keywords:** Congenital heart disease, Surgical planning, Imaging, Magnetic resonance imaging, 3D printing, Virtual reality, Computer aided design, Pediatric cardiology, Cardiothoracic surgery, Cardiac catheterization

## Abstract

**Background:**

Surgical and catheter-based interventions for congenital heart disease require precise understanding of complex anatomy. The use of three-dimensional (3D) printing and virtual reality to enhance visuospatial understanding has been well documented, but integration of these methods into routine clinical practice has not been well described. We review the growth and development of a clinical 3D modeling service to inform procedural planning within a high-volume pediatric heart center.

**Methods:**

Clinical 3D modeling was performed using cardiac magnetic resonance (CMR) or computed tomography (CT) derived data. Image segmentation and post-processing was performed using FDA-approved software. Patient-specific anatomy was visualized using 3D printed models, digital flat screen models and virtual reality. Surgical repair options were digitally designed using proprietary and open-source computer aided design (CAD) based modeling tools.

**Results:**

From 2018 to 2020 there were 112 individual 3D modeling cases performed, 16 for educational purposes and 96 clinically utilized for procedural planning. Over the 3-year period, demand for clinical modeling tripled and in 2020, 3D modeling was requested in more than one-quarter of STAT category 3, 4 and 5 cases. The most common indications for modeling were complex biventricular repair (*n* = 30, 31%) and repair of multiple ventricular septal defects (VSD) (*n* = 11, 12%).

**Conclusions:**

Using a multidisciplinary approach, clinical application of 3D modeling can be seamlessly integrated into pre-procedural care for patients with congenital heart disease. Rapid expansion and increased demand for utilization of these tools within a high-volume center demonstrate the high value conferred on these techniques by surgeons and interventionalists alike.

**Supplementary Information:**

The online version contains supplementary material available at 10.1186/s41205-022-00137-9.

## Introduction

Pediatric cardiothoracic surgery and catheter-based interventions depend on a precise understanding of complex and heterogeneous anatomy. Preoperative imaging is a critical tool for assessing the feasibility of complex repairs, as well as informing their optimal execution. 2-dimensional (2D) echocardiography is routinely utilized but relies on reader’s ability to reconstruct complex anatomy in 3-dimensions (3D) from 2D images. Cross-sectional imaging is often used to provide detailed anatomic information with high spatial resolution of the entire heart, but is traditionally viewed as a series of 2D slices which similarly rely upon the user to reconstruct the critical internal 3D anatomy. This limitation has led to the exploration of methods to more intuitively comprehend the latent 3D information in cross-sectional images by creating virtual and 3D printed models.

There is a plethora of literature describing the advantages of utilizing 3D modeling and visualization techniques for procedural planning in both pediatric and adult cardiology [1–11]. In patients with congenital heart disease much of the published data focuses on using 3D printing and more recently virtual or augmented reality, to inform surgical planning for patients with complex intracardiac anatomy such as double outlet right ventricle [12–16]. As a result of the improved visuospatial understanding of patient specific cardiac lesions conferred by viewing 3D representations of the anatomy, these 3D tools are heralded as beneficial adjunctive imaging tools that bring added-value to the preoperative planning space [17, 18]. However, the translation and scaling of these research methods to a routine clinical practice has not been well described.

In this work we describe the growth and development of a clinical 3D modeling service to inform pre-procedural planning within a large pediatric heart center.

## Materials and methods

### Patients

**Fig. 1:**
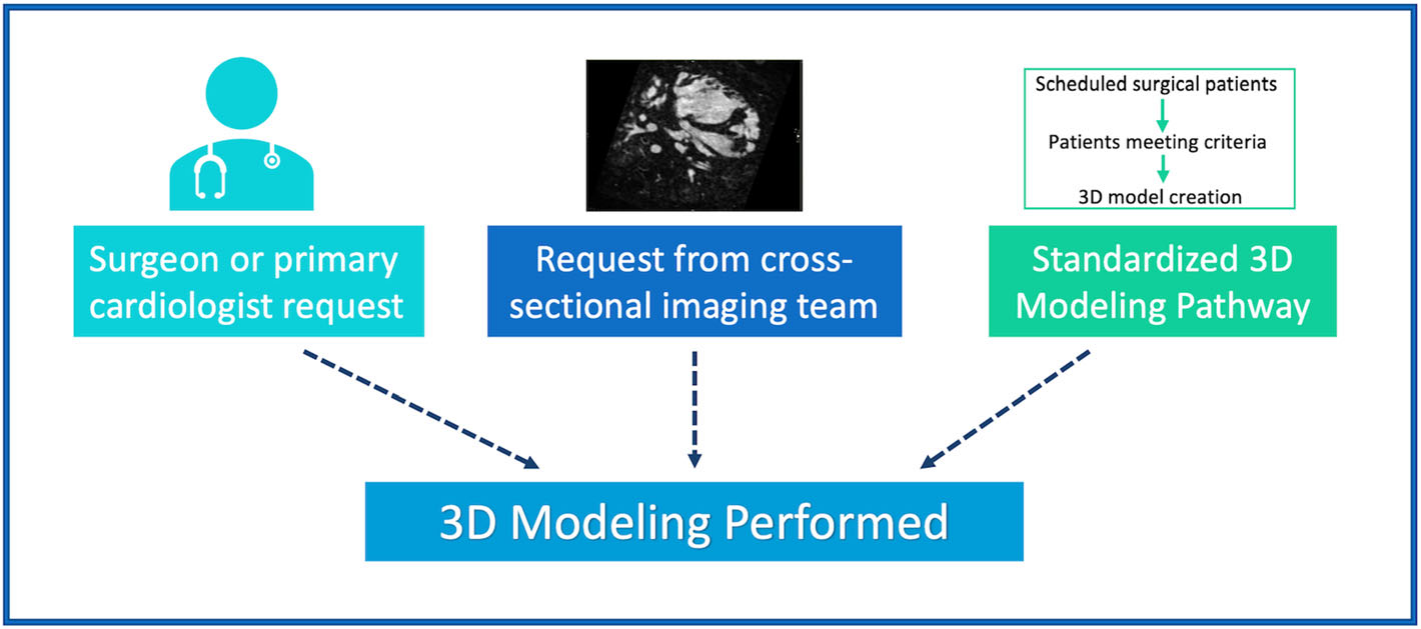
Case Selection for Clinical 3D Modeling

The institutional review board at the Children’s Hospital of Philadelphia approved this study. We reviewed 36 months of operations of the clinical 3D printing service (from 2018 through 2020), from which requisitions were received as follows:

1) Request of the primary cardiologist or surgeon 2) request of the cross-sectional imaging team and/or 3) meeting procedural criteria as part of a standardized 3D modeling pathway (Fig. 1). Through the standardized 3D modeling pathway, patients undergoing one of a specific set of procedures AND had high-resolution cross-sectional imaging, would routinely have 3D modeling performed. The procedure-based criteria for this pathway were developed organically over a 12-month period (in 2018). Ultimately, the following procedures were integrated into the modeling pathway in January of 2019: 1) Multiple muscular ventricular septal defects (VSD) 2) Determination of feasibility of biventricular repair 3) Tetralogy of Fallot / Pulmonary Atresia with Major Aortopulmonary Collaterals (TOF PA MAPCA) 4) Transcatheter Innominate Vein Turndown.

### Image acquisition

The 3D printing program included source imaging by Cardiac Magnetic Resonance (CMR), all of which were performed on a 1.5 Tesla scanner (Magnetom Avento, Siemens Healthcare) with either gadobutrol (*Gadavist, Bayer HealthCare, New Jersey)* of ferumoxytol *(Feraheme, AMAG Pharmaceuticals, Massachusetts)* as the contrast agent. Retrospective imaging studies were deemed to be adequate for 3D modeling purposes if there was a cardiac gated magnetic resonance angiography (MRA) sequence performed with a maximum voxel size of 1.2 mm^3^. Computed tomography (CT) studies were performed on a SOMATOM Definition Flash (Siemens Healthcare) with an iodinated contrast agent (*Iohexol, Omnipaque, GE Healthcare Inc.)*. Similarly, studies were deemed adequate if CT angiography was acquired with a voxel size ≤1mm^3^.

Prospective image acquisition for the purpose of 3D modeling entailed a ferumoxytol-enhanced CMR with a navigated cardiac inversion recovery FLASH sequence in both systole and diastole. The maximum interpolated voxel size for patients less than 10 kg was 0.9mm^3^ and 1.2mm^3^ for patients ≥10 kg.

In addition, regardless of modality, digital or printed models were not created for patients for whom there was any imaging artifact in the anatomic region of interest.

### Image segmentation

Selected digital imaging and communications in medicine (DICOM) files were imported into FDA cleared dedicated 3D printing [1] software (*Mimics Innovation Suite version 20, Materialise, Belgium)*. Semi-automatic segmentation techniques were used to create masks and subsequent 3D volumes of the blood pool and if indicated, the myocardium, semilunar valve annuli and atrioventricular (AV) valve annuli (Supplemental Data: Fig. 1). The 3D volumes were post processed to create a hollow blood pool with uniform thickness ≤ 1.0 mm, that was then merged with the myocardial volume to create a whole heart model. The post-processed 3D volumes were used to create stereolithography (STL) files. When clinically indicated, both systolic and diastolic models were created for a single case.

All segmentation and post-processing were performed by an advanced imaging cardiology fellow (RMG) or clinical engineer (ES). Prior to clinical use, digital models were verified for anatomic accuracy within the region of interest, by the advanced imaging fellow and/or cardiac MRI attending.

### 3D visualization tools for procedural planning

#### 3D printing

**Fig. 2:**
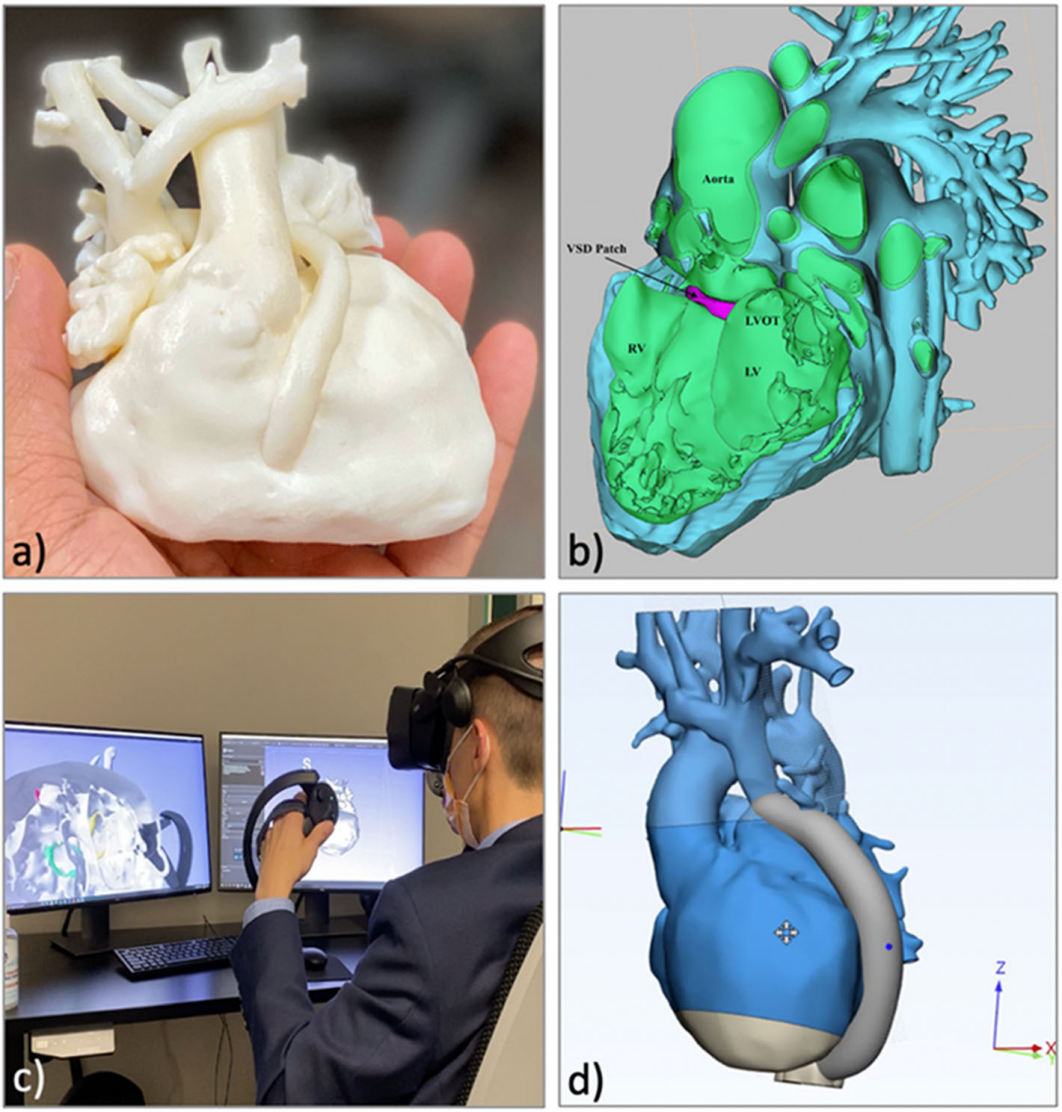
3D Visualization Modalities. **a**) 3D printed model; **b**) Digital model viewed using a 3D PDF; **c**) Virtual Reality; **d**) CAD modeling of digital repairs. *3D = Three-dimensional; PDF = portable document file; CAD = computer aided design*

3D printing was performed by the Children’s Hospital Additive Manufacturing for Pediatrics (CHAMP) Lab, a central 3D printing lab administered by the Department of Radiology at CHOP. A material jetting printer, (Objet500 Connex 3 (prior to July 2019) and J750, Stratasys, Eden Prairie, MN), was used to create physical models at 100% scale. Prior to 2020, models were created using rigid photopolymers with a minimum thickness of 1.0 mm. After January 2020, the option to print models with a minimum thickness of 0.75 mm and using flexible rubber photopolymers was available (Fig. 2a, Supplemental Fig. 2).

#### 3D PDF / flat screen visualization

The generated STL files were visualized on a flat (2D) screen (Fig. 2b) using one of two methods. 1) The files were converted to a 3D portable document file (PDF) format and viewed using a free Adobe Acrobat Reader (*Adobe Systems, San Jose CA)*. 2) Starting in October 2020, the ability to view STL files using a recently developed Dynamic Modeling Module [19] in 3D Slicer (www.slicer.org) was available.

#### CAD-based modeling

Standard CAD operations were employed using 3Matic software (*Materialise, Belgium*) to digitally design surgical repair options when clinically indicated. Sweep-loft functions were used to design Fontan and right ventricle to PA conduits, as well as simulate interposition grafts. Simple VSD patches were created according to the Radiologic Society of North America 2015 primer [20]. Intracardiac baffles were designed using a combination of spline creation, sweep-loft and hollow functions.

With the release of the Baffle Tool in the SlicerHeart extension for 3D Slicer (www.slicer.org), all intracardiac baffle design was performed using this software due to its simplicity and superior functionality. Description of this modelling tool has been published previously [19] (Fig. 2d).

#### Virtual reality

The STL files were visualized in virtual reality using the SlicerVR module in 3D Slicer (www.slicer.org) running on a standard PC with a RTX 2080TI graphics processor and a Valve Index Headset (*Valve Corporation Bellevue, WA*) [21] (Fig. 2c).

#### 3D modality utilization

Utilization of the above-mentioned four 3D modalities was based on both availability of the technique and proceduralist request. From program inception through July 2019 the only available visualization modalities were 3D printing and digital 3D PDF. In July of 2019, CAD-based modeling of surgical repairs as well as the ability to view STLs in virtual reality became available.

### Facilities

**Fig. 3:**
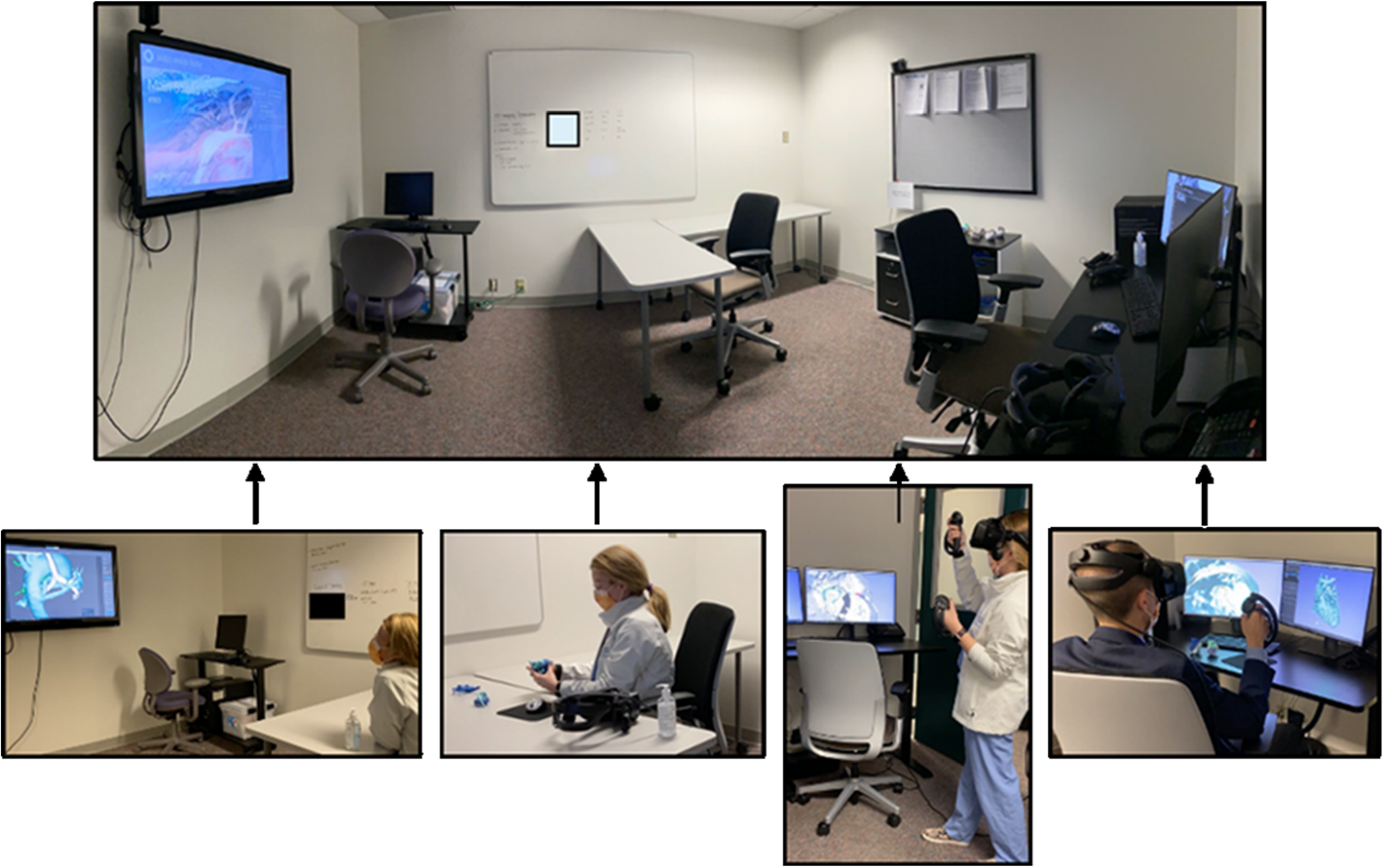
Dedicated 3D Imaging Review Suite. Depiction of the 8′ × 15′ 3D Imaging Review Suite, with dedicated space for reviewing patient anatomy in digital formats, printed models and in virtual reality

An 8 ft. × 15 ft. room was designated by the cardiac center for image review (Fig. 3). A computer with two monitors was used for interactive work with a remote wall mounted HD TV for group review. Two SteamVR 2.0 Base Stations (*Valve Corporation Bellevue, WA*) were mounted on opposite sides of the room. The majority of the room’s footprint was left open to allow ease of movement when utilizing the VR system. The room was situated between the interventional cardiology and cardiothoracic surgery office suites, allowing for proximity to the proceduralists.

### Clinical review

Images and models were reviewed in multiple forums and formats. Physical and/or on-screen models were presented at the weekly cardiothoracic surgical conference. The 3D modelling was reviewed in further detail with a small group of case-specific multidisciplinary team members, using all available 3D visualization formats. At these meetings, all surgical approach options were discussed and visualized using the models. Afterwards, 3D PDF or screen recording of model manipulation was sent to all team members for reference.

## Results

### Modeling service utilization

From 2018 to 2020 there were 112 individual 3D modeling cases performed in the Cardiac Center at The Children’s Hospital of Philadelphia. Of these, 16 cases were performed for educational purposes and the remaining 96 were utilized clinically to inform surgical and/or procedural planning. A total of 127 individual models were created for these 96 clinical cases.

**Fig. 4:**
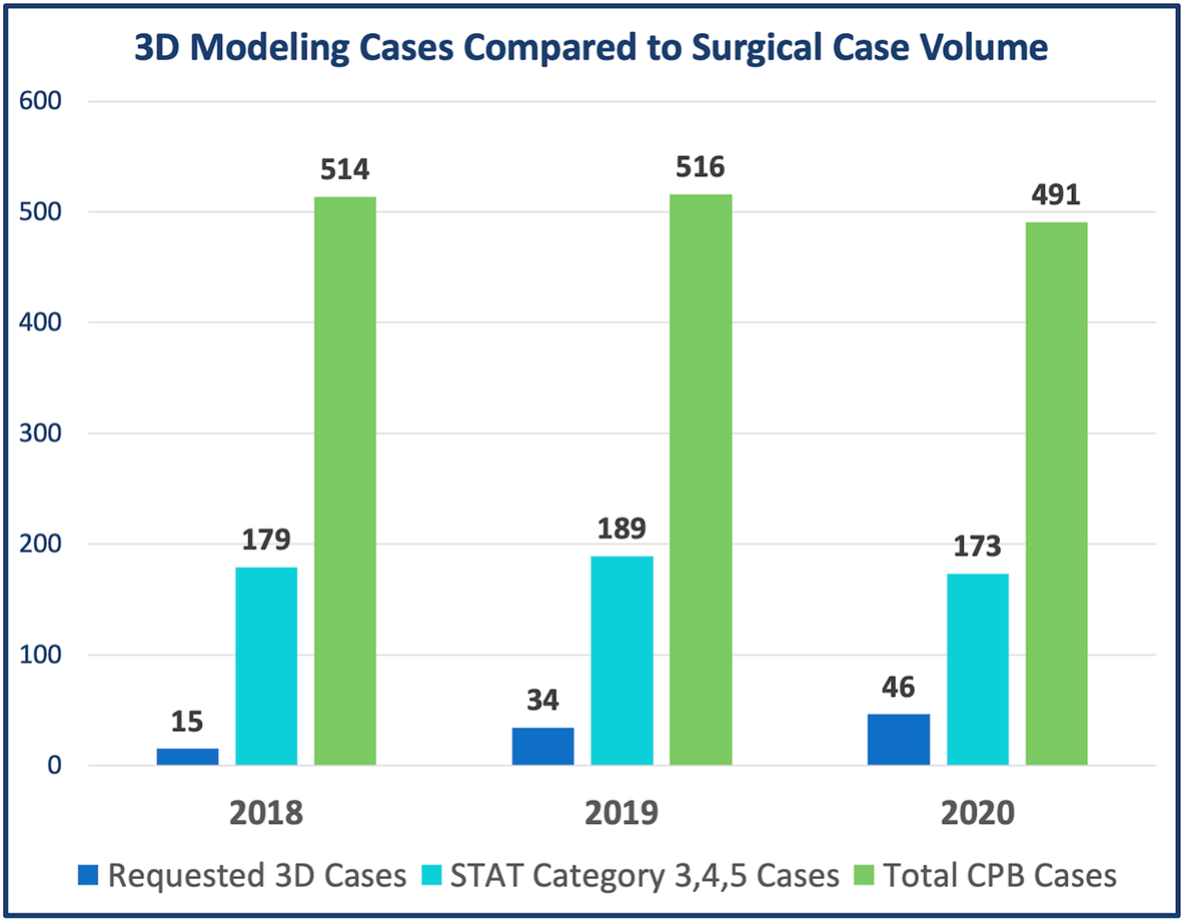
Number of 3D Modeling Cases Compared to Surgical Case Volume. *3D = Three-dimensional; STAT = The Society of Thoracic Surgeons and The European Association for Cardio-Thoracic Surgery; CPB = cardiopulmonary bypass*

Over the 3-year period, there was an annual increase in the number of 3D modeling cases performed, from 15 unique cases modeled in 2018 to 47 cases in 2020. Compared to the number of complex / increased risk surgeries performed as represented by the annual total of STAT (The Society of Thoracic Surgeons and The European Association for Cardio-Thoracic Surgery) Category 3, 4 and 5 cases conducted at our institution, this represents an increase in utilization of 3D modeling from 8% of the highest risk cases in 2018 to 27% in 2020 (Fig. 4).

### Origination of request for modeling

**Fig. 5:**
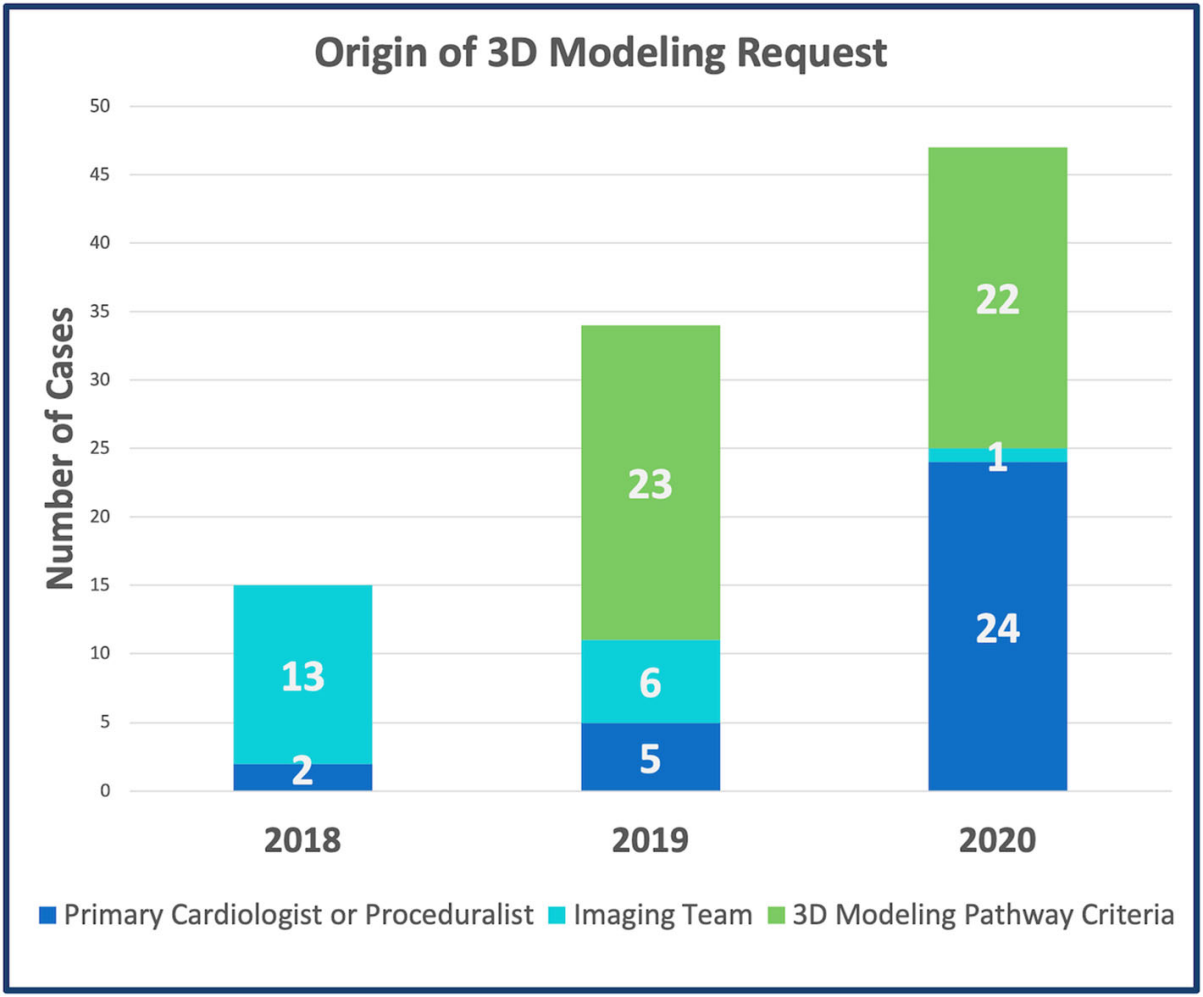
Origin of 3D Modeling Request

In 2018, the majority (87%) of requests for 3D modeling originated from the cross-sectional imaging team. In 2019 and 2020, clinical 3D modeling was performed predominantly due to request of the primary team or the patient met procedural criteria for the standardized 3d modeling pathway (Fig. 5).

### Distribution of anatomic and procedural indication for modeling

Of the 96 clinical 3D cases modeled during this time, nearly one-third of the patients (*n* = 30, 31%) had modeling performed to evaluate intracardiac anatomy for a potential complex biventricular repair (Table 1). The 2nd most common reason for 3D modeling was preoperative evaluation of extracardiac pathology (*n* = 19, 20%) such as anomalous systemic or pulmonary venous connections, followed by proposed repair of multiple ventricular septal defects (*n* = 11, 12%) and MAPCA unifocalization (*n* = 10, 11%). The remaining indications included complex Fontan planning, ventricular assist-device fit testing, transcathether innominate vein turndown and residual disease/consideration of revision of prior surgical repair. The variety of indications for 3D modeling increased over time, but with evaluation for complex biventricular repair remaining the most common (Table 2).

**Table 1:** Description of clinical 3D modeling cohort

	Age^a^ at request (years)	Cross-sectional imaging modality
All patients (*n* = 96)	2.4 (0.85, 6.1)	19% CTA / 81% CMR
*Single ventricle* vs *biventricular repair* *(n = 30)*	0.72 (0.44, 1.6)	13% CTA / 87% CMR
*Complex extracardiac anatomy (n = 19)*	6.2 (2.5, 16.4)	5% CTA / 95% CMR
*Multiple VSDs (n = 11)*	1.3 (0.77, 2.6)	9% CTA / 91% CMR
*Unifocalization (n = 10)*	0.45 (0.30, 0.84)	100% CTA
*Complex Fontan Planning (n = 8)*	2.7 (2.2, 4.1)	100% CMR
*VAD Planning (n = 6)*	7.1 (3.0, 11.3)	50% CTA / 50% CMR
*Revision of Repair (n = 6)*	2.1 (0.8, 5.5)	17% CTA / 83% CMR
*Inn Vein Turndown (n = 6)*	12.4 (7.2, 14.0)	100% CMR

*3D* Three-dimensional, *CTA* computed tomography angiography, *CMR* cardiac magnetic resonance imaging, *VSD* ventricular septal defect, *VAD* ventricular assist device, *Inn Vein* Innominate Vein

^a^Age presented as median and interquartile range

**Table 2:** Anatomic or procedural indication for modeling request according to year

Indication for modeling	2018 (*n* = 15)	2019 (*n* = 34)	2020 (*n* = 47)
*Biventricular Repair Feasibility*	2 (13%)	13 (38%)	15 (32%)
*Unifocalization*^a^	0	7 (21%)	3 (6%)
*Multiple VSDs*	1 (7%)	3 (9%)	7 (15%)
*Complex Vascular Anatomy*	5 (33%)	6 (18%)	8 (17%)
*Complex Fontan Planning*	5 (33%)	0	3 (6%)
*VAD Planning*	0	1 (3%)	5 (11%)
*Revision of Prior Surgical Repair*	2 (13%)	3 (9%)	1 (2%)
*Innominate Vein Turndown*	0	1 (3%)	5 (11%)

Results expressed as count (percentage)

*VSD* ventricular septal defect, *VAD* ventricular assist device

^a^Refers to unifocalization of aortopulmonary collaterals

### Retrospective vs prospective 3D modeling

The majority of 3D modeling cases were requested and/or met indication for modeling retrospectively (*n* = 64, 67%). In these cases, the cross-sectional imaging used for the creation of the 3D models was already acquired when the 3D team was made aware of the modeling request. In 2019 and 2020, 33 cases were requested *prior* to the patient undergoing cross-sectional imaging, and image acquisition was optimized for 3D modeling (Table 3).

**Table 3:** Number of prospective and retrospective cases according to year

	Prospective cases	Retrospective cases
*2018*	0	15
*2019*	15	19
*2020*	17	30

### Utilization of 3D visualization modalities

**Fig. 6:**
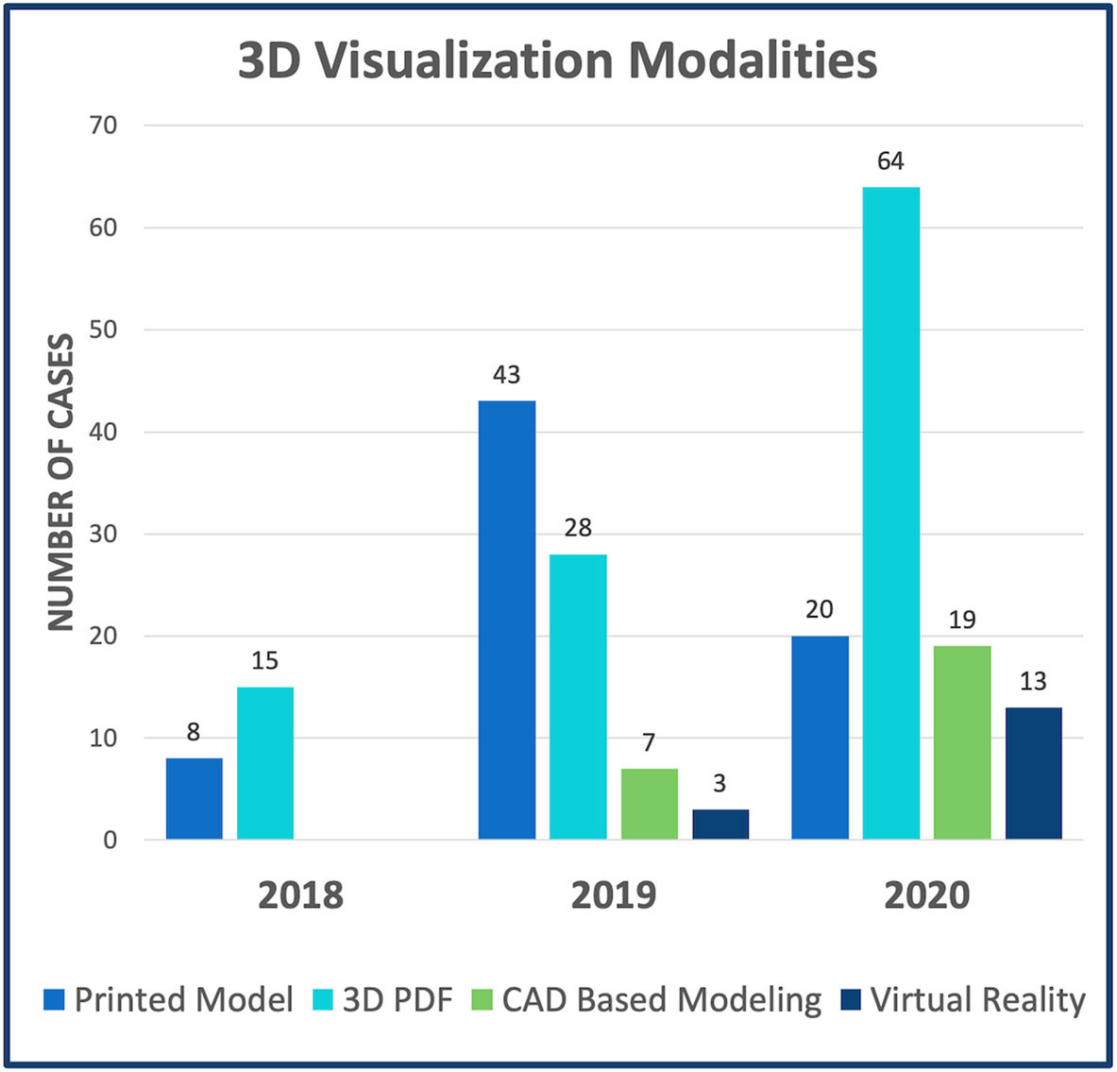
Utilization of 3D Visualization Modalities By Year. *3D = Three-dimensional; PDF = portable document file; CAD = computer-aided design*

In the first year of the 3D modeling program, all cases were presented as printed models with flat-screen model viewing using a 3D PDF. Over the following 2 years (2019 and 2020) there was increased utilization of new 3D visualization formats, such as CAD based modeling of potential surgical repairs and model viewing in virtual reality. There was an accompanying decrease in the use of printed models (Fig. 6).

## Discussion

We present a retrospective review of over 100 3D modeling cases performed at a single pediatric cardiothoracic surgical program over the span of 3 years. To our knowledge this is one of the first published descriptions of a clinical 3D modeling service that is integrated into routine pre-procedural care of pediatric patients with acquired or congenital heart disease. To be clear, the intent of this manuscript is to describe *how* such a program can be developed and incorporated into clinical care. Outcomes-based research on 3D visualization for various procedures is outside the scope of this work.

### Adoption and integration of clinical 3D modeling service

The steady increase in service volume, modeling requests and prospective imaging studies all demonstrate the rapid adoption and utilization of 3D modeling for procedural planning at our institution. Most notably, while the majority of cases modeled during the first year of the program were performed at the request of the cross-sectional imaging team, by the 2nd and 3rd years there was a shift toward proceduralist and clinician-initiated request for 3D modeling. In the most recent year, at the least two-thirds of the cases that met criteria for modeling had a request placed by a member of the primary cardiac team. In addition, the increase in prospective (pre-image acquisition) requests suggests that surgeons, interventionalists and cardiologists are integrating 3D modeling tools into their procedural planning and want to ensure that these tools would be available to their patient. Prospective requests also allow the imaging team to ensure adequate spatial and temporal resolution of the region of interest, improving the quality of the raw data. Though it was unavailable during the time period evaluated in this review, at the time of manuscript preparation clinical orders for 3D model creation have been incorporated into the electronic medical record (*Epic Systems Corporation, Verona WI)*.

### Evolution of 3D modeling tools

Growth of the 3D modeling clinical service was not limited to the volume of cases, but also encompassed technological advancements in modeling techniques. Over the course of 3 years, there was a shift from the use of static digital or printed 3D models to interactive 3D representations. This was driven by progress in both the additive manufacturing and gaming industries. The acquisition of a newer generation printer (*J750, Stratasys, Eden Prarie MN*) with the capability to accurately print structures as thin as 0.014 mm with flexible, rubber-like materials (*Agilus 30, Stratasys, Eden Prarie MN*) facilitated interaction with the printed models (Supplemental Fig. 2, Additional files 3 and 4: Supplemental Video). It gave surgeons the ability to trial ventriculotomy sites, assess exposure of VSDs through the tricuspid valve and determine distance and geometry of anomalous vessels in patients undergoing anomalous pulmonary venous drainage repair.

There was concurrent advancement in digital and virtual modeling. With the development of the SlicerVR module in 3D slicer, the STL files used to create printed models could be imported into the virtual space within minutes. After the first case was demonstrated, there was rapid integration of VR into the modeling program, driven predominantly by surgeon and interventional team requests. Simultaneously there was also increased application of CAD-based modeling of potential surgical repairs. Intracardiac baffles, Fontan conduits, interposition grafts simple VSD patches and other structures were easily designed using a combination of proprietary and open-source tools. This facilitated evaluation of potential repairs with regard to the surrounding structures in the region of interest, as well as visualization of residual lesions (i.e., LVOT obstruction after LV to aorta baffle placement or compression of an RV-PA conduit beneath the sternum).

### Knowledge gained through program development

Development of the 3D modeling program was guided by the iterative efforts to define and refine the type of cases for which 3D modeling was most impactful. This occurred through multidisciplinary efforts and consistent post-procedural discussions with the surgeons and interventionalists. Predictably, nearly one-third of all modeling cases were performed for evaluation of complex biventricular repair feasibility. The most commonly published descriptions of 3D printing in congenital heart disease are of patients with double outlet right ventricle, [12–16, 19, 22] and in this cohort we found similar utility in creating models for patients with other conotruncal anomalies and similar need for an intracardiac baffle. In addition, based on proceduralist feedback and number of requests, it was evident that 3D modeling was impactful for planning transcatheter innominate vein turndowns, closure of multiple muscular VSDs and “non-traditional” Fontan conduit placement.

Two key elements in the workflow that enhanced feasibility of 3D modeling as a clinical service were image resolution guidelines and physical proximity of the VR and modeling space. In the first year of the program, we learned that creating models from source imaging that has low spatial resolution, artifact in the region of interest or was acquired in an unsuitable phase of the cardiac cycle all led to increased segmentation times and decreased model quality. By setting guidelines for acceptable resolution and quality of cross-sectional imaging, we improved efficiency and detail of model generation. Furthermore, in 2019 the establishment of dedicated 3D imaging space located in close proximity to the proceduralists facilitated increased communication and collaboration, increased use of the 3D visualization tools and improved awareness of the ongoing developments in modeling techniques.

### Challenges

Image segmentation and CAD modeling is a time-consuming process requiring a highly skilled user. Currently modeling is not directly reimbursed by traditional clinical mechanisms. The development of this program was largely the result of the dedication of a single individual during fellowship training. Facilities and resources were obtained through a competitive institutional grant. There are ongoing efforts aimed at securing reimbursement, most notably the four American Medical Association-approved Category III Current Procedural (CPT) codes for 3D printed models and patient specific surgical guides which serves to track procedural use [23]. Until those are transitioned to Category I Codes, scaling and longitudinally supporting such a program will require creative funding mechanisms. Yet in our experience, the surgeons and interventionalists found 3D modeling so compelling that requests exponentially increased over time. As such, we believe that modeling may become an integral part of planning complex procedures, and accepted as the “cost of doing business” for large congenital heart centers.

Demonstrating direct impact of 3D modeling on surgical outcomes is difficult due to the multifactorial nature of traditional post-operative metrics (i.e., cardiopulmonary bypass time, ICU length of stay), and difficulty of performing a conclusive randomized trial in this setting. However, techniques such as computational fluid dynamics and 4D flow CMR can illustrate patient specific changes in flow patterns and hemodynamics, that could potentially facilitate objective and concrete evaluation of the effect of 3D modeling on surgical outcomes.

### Limitations

A limitation of this manuscript is that until 3D modeling of patient anatomy and surgical repairs are approved for reimbursement, funding mechanisms to support this type of program will vary between institutions. Ultimately this will affect the size and capacity of the established clinical service.

### Future work and evolution of modeling

Further creation of specialized tools for modeling will increase the speed and accuracy of modeling. Given the unique needs of modeling congenital hearts custom application design may be needed. Innovative techniques [24, 25] such as machine learning may inform this process and make it more rapid and allow for users with less experience. Focused studies on the comparative benefits of the various 3D visualization techniques are currently ongoing.

## Conclusions

Clinical application of an evolving armamentarium 3D modeling tools is feasible within large pediatric cardiothoracic surgical program. Through a multidisciplinary approach these techniques can be seamlessly integrated into pre-procedural care, efficiently providing children with extremely complex cardiac pathology access to the most advanced diagnostic imaging tools. Increased application of 3D modeling will continue to spur technological advancement and innovation. Future work should focus on the development of new techniques to improve the ease and speed with which 3D modeling technologies can be employed.

## Supplementary Information

**Additional file 1: Supplemental Fig. 1.** DICOM to Image Segmentation. DICOM = Digital Imaging and Communications in Medicine.

**Additional file 2: Supplemental Fig. 2.** 3D Printed Models. **a**) Patient-specific model demonstrating Tetralogy of Fallot Pulmonary Atresia with Major Aortopulmonary Collaterals; printed with material jetting (Connex Objet500, Stratasys, Eden Prairie MN; TangoPlus). **b**) Patient-specific model demonstrating RV to Aorta anatomy. Pink = aortic annulus; Green = tricuspid annulus. Printed with material jetting (J750, Stratasys, Eden Prairie MN; Agilus30 and VeroVivid) . **c**) Patient-specific model created in flexible material, demonstrating thickness of the vessel wall (0.75 mm). Printed with material jetting (J750, Stratasys, Eden Prairie MN; Agilus30).

**Additional file 3: Video 1.** Overview of Clinical 3D Modeling Program.

**Additional file 4: Video 2.** Clinical Case Example.

**Additional file 5: Figure.** Visual Abstract.

## Data Availability

The imaging data used and analyzed during this study are available from the corresponding author on reasonable request.

## Abbreviations

2D: 2-dimensional
3D: 3-dimensional
AV: Atrioventricular
CAD: Computer Aided Design
CMR: Cardiac Magnetic Resonance
CT: Computed Tomography
STAT: The Society of Thoracic Surgeons and The European Association for Cardio-Thoracic Surgery
STL: Stereolithography
TOF PA MAPCA: Tetralogy of Fallot / Pulmonary Atresia with Major Aortopulmonary Collaterals
VSD: Ventricular Septal Defect
VR: Virtual Reality
