# Supplementary figures and images for: Clinical 3D modeling to guide pediatric cardiothoracic surgery and intervention using 3D printed anatomic models, computer aided design and virtual reality
Source: 3D Print Med. 2022 Apr 21;8:11. doi: 10.1186/s41205-022-00137-9 (PMC9027072; doi:10.1186/s41205-022-00137-9)

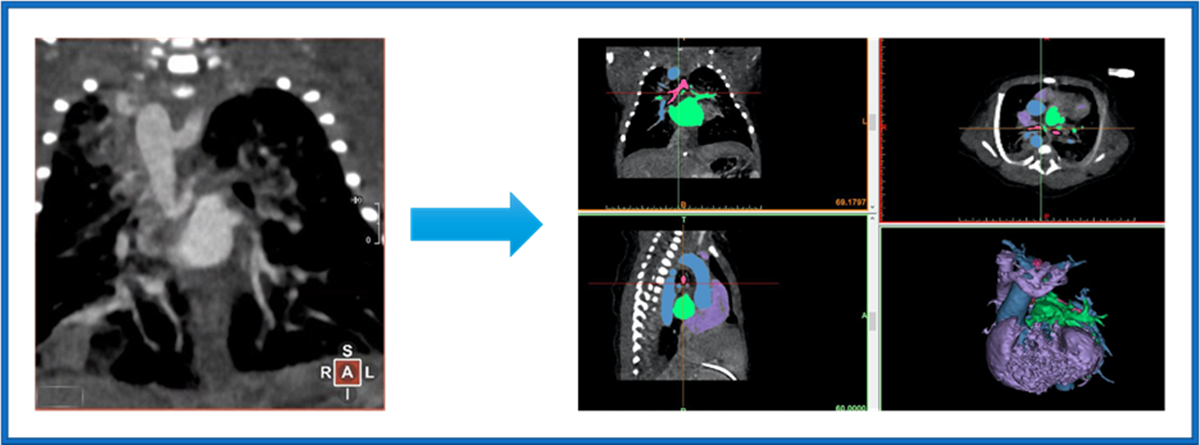

Supplement: Supplementary file 1 — Additional file 1: Supplemental Fig. 1. DICOM to Image Segmentation. DICOM = Digital Imaging and Communications in Medicine. [file 41205_2022_137_MOESM1_ESM.tif]

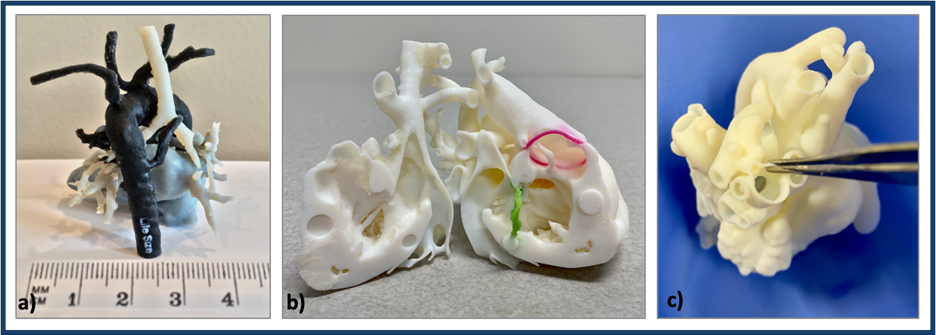

Supplement: Supplementary file 2 — Additional file 2: Supplemental Fig. 2. 3D Printed Models. a) Patient-specific model demonstrating Tetralogy of Fallot Pulmonary Atresia with Major Aortopulmonary Collaterals; printed with material jetting (Connex Objet500, Stratasys, Eden Prairie MN; TangoPlus). b) Patient-specific model demonstrating RV to Aorta anatomy. Pink = aortic annulus; Green = tricuspid annulus. Printed with material jetting (J750, Stratasys, Eden Prairie MN; Agilus30 and VeroVivid) . c) Patient-specific model created in flexible material, demonstrating thickness of the vessel wall (0.75 mm). Printed with material jetting (J750, Stratasys, Eden Prairie MN; Agilus30). [file 41205_2022_137_MOESM2_ESM.tif]

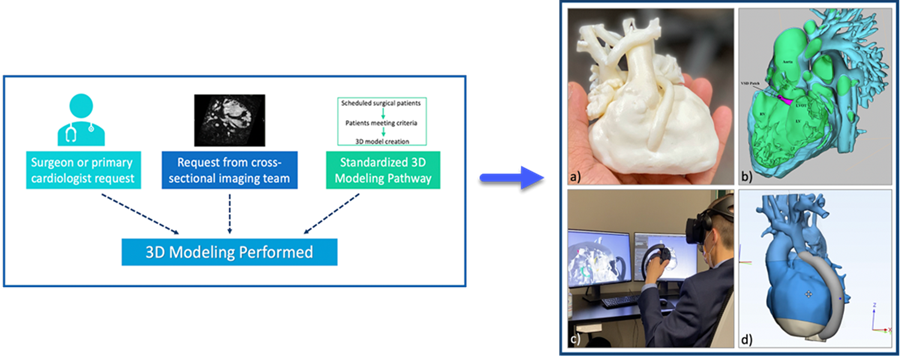

Supplement: Supplementary file 5 — Additional file 5: Figure. Visual Abstract. [file 41205_2022_137_MOESM5_ESM.tif]
